# ^18^F-FDG PET/CT in Pleural Epithelioid Hemangioendothelioma

**DOI:** 10.22038/aojnmb.2016.7971

**Published:** 2017

**Authors:** Domenico Albano, Giovanni Bosio, Andrea Tironi, Francesco Bertagna

**Affiliations:** 1 Nuclear Medicine, University of Brescia and Spedali Civili Brescia, Brescia, Italy; 2 Department of Molecular and Translational Medicine, Anatomic Pathology Section, Spedali Civili Brescia, Brescia, Italy

**Keywords:** 18F-FDG PET/CT, Pleural epitheliod hemangioendothelioma

## Abstract

Pleural epithelioid hemangioendothelioma (EHE) is a rare malignancy of vascular-endothelial origin with non-specific symptoms and an unpredictable outcome. Diagnosis of this condition by imaging modalities is challenging, and no standard therapeutic approaches have been established in this regard. In this paper, we described the case of a patient with a low-grade fever, coughing and chest pain who underwent ^18^F-FDG PET/CT after a positive thorax CT showing multiple bilateral calcified pulmonary nodules and extensive right-sided pleural effusion. Moreover, PET/CT revealed increased tracer uptake on the nodular pleural thickening and one nodule in the upper lobe of the right lung. A diagnostic thoracentesis was performed to obtain the pleural fluid. However, cytology was not diagnostic, and the subsequent thoracotomy with pleural fluid drainage and pleural biopsy was positive for pleural EHE. The study showed also an abundant non-FDG-avid pleural effusion in the collapsed right lung. Despite chest tube insertion and partial drainage of the volume, patient’s condition deteriorated, and patient passed away six months after the PET scan.

## Introduction

Epithelioid hemangioendothelioma (EHE) is a rare malignancy of vascular origin, which may occur in different locations such as the lungs, liver and bones. Etiology of EHE remains unknown. In the current literature, EHEs of the pleural origin have been less frequently described than those arising in other sites. Additionally, these tumors are reported to be more aggressive comparatively (1-4).

In this paper, we described a unique case of pleural EHE extending to the lungs in a 76-year-old female patient. To the best of our knowledge, this is the third reported case of EHE of the pleural origin with positive ^18^F -FDG PET/CT. In the present case, ^18^F-FDG PET/CT contributed to the recognition of nodular pleural thickening uptake, which resulted in the diagnosis of pleural EHE.

## Case report

A 76-year-old female patient was referred with a history of breast cancer treated with quadrantectomy, chemotherapy and radiotherapy 10 years before. She was a non-smoker. Due to the occurrence of dyspnoea, coughing and chest pain, the patient underwent chest computerized tomography (CT), which revealed a nodular pleural thickening in the right lung, including an extrapleural lesion on the upper lobe of the right lung (diameter: 2 cm), multiple calcified nodules in both lungs, and extensive right-sided pleural effusion.

Subsequent ^18^F-FDG PET/CT was performed for malignancy suspicion. Moreover, PET/CT was acquired 60 minutes after the intravenous injection of ^18^F-FDG (3.5 MBq/kg) on Discovery 690 tomograph (GE-Milwaukee, Wi, USA; 64-slice CT, 80 mA, 120 Kv; 2.5 min/bed; 256×256 matrix, 60 cm field of view).

**Figure 1:**
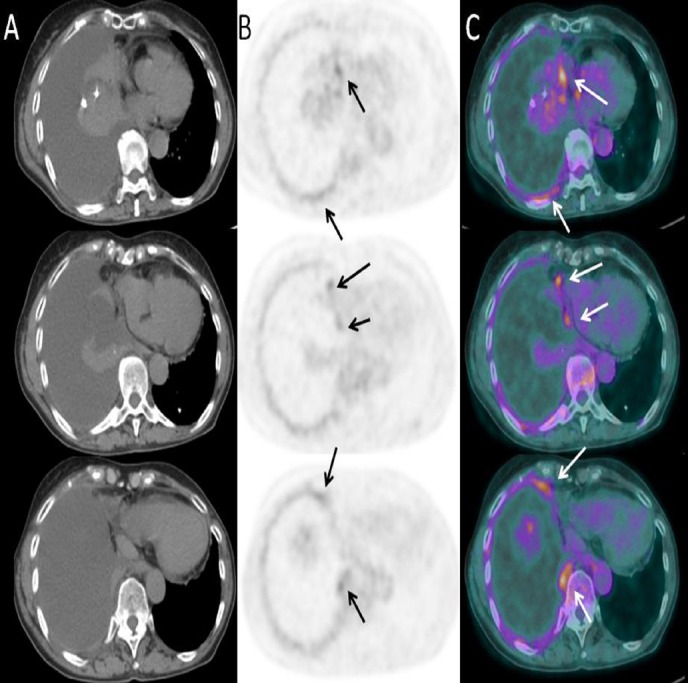
Three consecutive axial CT (A), PET (B) and fused PET/CT (C) images showing moderate FDG uptake in nodular pleural thickening of right lung (SUV_max_ 4.6) with non-FDG-avid pleural effusion

**Figure 2:**
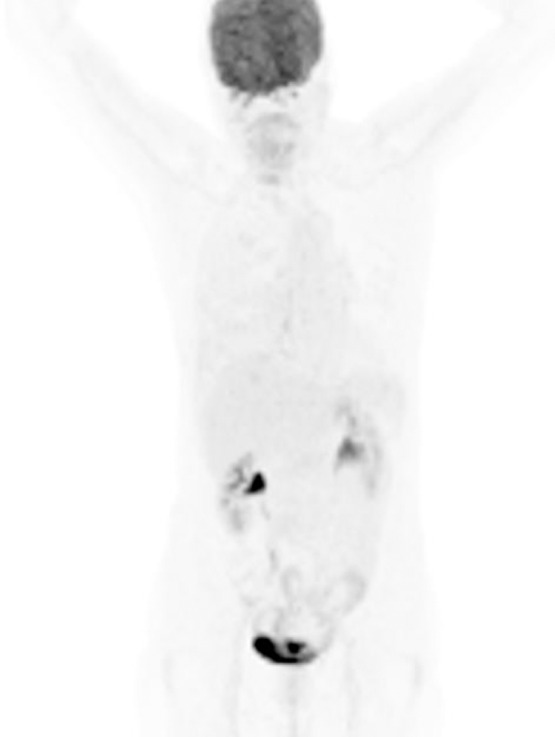
Maximum intensity projection (MIP) showing no other pathological FDG uptake in body (except previously described pleural lesions)

Glucose level of the patient was 113 mg/dl, and ^18^F-FDG-PET/CT showed diffuse FDG accumulation in the right pleural thickening (Figure 1). Whole-body images revealed no other FDG-avid abnormalities, and it could not detect all the nodules identified in CT-scan (Figure 2).

**Figure 3:**
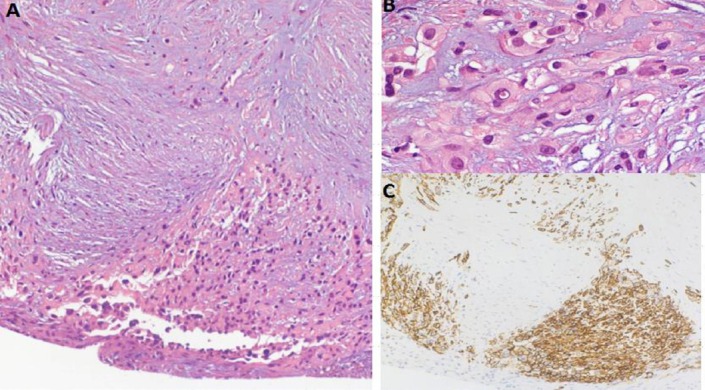
Thoracoscopic pleural biopsy showing epithelioid hemangioendothelioma invading pleura HE×100 (A); HE×400 (B); CD34 reactivity of neoplastic epithelioid cells, anti-CD34×100 (C)

FDG-PET findings showed a high suspicion of malignancy. Diagnostic thoracentesis was conducted to obtain the pleural fluid, while cytology was not diagnostic. Subsequent thoracotomy with pleural fluid drainage and pleural biopsy confirmed pleural EHE (Figure 3).

Pleura were characterized by the fibrous thickening areas, in which epithelioid cells were observed. These cells were detected in an isolated form, in small groups, or focally in large aggregates invading downward from the surface. Alternating to mesothelial cells on the surface, neoplastic epithelioid cells were detected as well.

Neoplastic cells were epithelioid with round nucleous and occasionally appeared pleomorphic with evident nucleolus and lightly eosinophilic cytoplasm, which had cytoplasmic vacuoles containing red blood cells in some cases. In immunohistochemical stains, neoplastic cells reacted to vascular markers (CD31 and CD34), while they were negative to mesothelial markers (panCK, AE1/AE3, Ck 5/6, EMA, calretinin, WT1), TTF1, Napsin, and estrogen receptors.

Despite chest drainage, patient’s condition deteriorated rapidly, and she died due to respiratory failure six months after the PET scan.

## Discussion

EHE is a rare type of malignancy, which is of vascular-endothelial origin. This tumor mainly develops in the lungs, bones, liver and soft tissues. In most cases, only a single organ is involved at presentation.

Pleural EHE is rare, usually affects men and is associated with poor prognosis with disseminated disease (1-4). Mean age of the patients at presentation is 45 years, with a female-to-male ratio of 1:2.3 and mean survival rate of 10 months. Patients with EHE present with a heterogeneous array of symptoms, the most common of which are chest pain, dyspnea and coughing.

Radiographically, diagnosis of pleural EHE is nonspecific and can be easily mistaken for malignant mesothelioma or inflammatory diseases (e.g., pleural infections). In chest CT-scan, pleural effusion is frequently observed with localized or diffuse nodular pleural thickening, which is similar to the current case (5, 6).

Diagnosis of EHE could be confirmed through the pathological examination of the specimen obtained by surgical lung biopsy. On immunohistochemistry, EHEs show the typical phenotype of endothelial cells with reactivity to vascular-specific markers, such as factor VIII-related antigen, CD31, CD34, and Fli-1 (6).

To date, no consensus has been reached regarding the effective management of EHEs. If the disease is limited, surgical resection of the tumor might be considered the best choice. Frequently, extension of the disease at diagnosis precludes the utility of surgery, which is similar to the present case.

Various chemotherapy regimens have been applied in this regard, including carboplatin, etoposide, paclitaxel, gemcitabine, doxorubicin, and interferon, yielding unsatisfactory results. In the current literature, about 30 cases have been classified and described as pleural EHE (5-20), while only one patient has received ^18^F-FDG PET/CT with a moderate FDG uptake (19).

Despite the limitations of the current study, this article may contain important clinical implications on ^18^F-FDG PET/CT in pleural EHE, especially regarding the possible role of PET/CT in the proper staging and management of these patients. To the best of our knowledge, this is the second available article in the literature to document a rare case of histologically confirmed pleural EHE, characterized by FDG accumulation on ^18^F-FDG PET/CT.
